# 2-Chloro-*N*-[4-chloro-2-(2-chloro­benzo­yl)phen­yl]acetamide

**DOI:** 10.1107/S1600536810003375

**Published:** 2010-01-30

**Authors:** Grzegorz Dutkiewicz, B. P. Siddaraju, H. S. Yathirajan, B. Narayana, Maciej Kubicki

**Affiliations:** a Department of Chemistry, Adam Mickiewicz University, Grunwaldzka 6, 60-780 Poznań, Poland; b Department of Chemistry, V. V. Puram College of Science, Bangalore 560004, India; c Department of Studies in Chemistry, University of Mysore, Manasagangotri, Mysore 570 006, India; d Department of Studies in Chemistry, Mangalore University, Mangalagangotri 574 199, India

## Abstract

In the title compound, C_15_H_10_Cl_3_NO_2_, an intra­molecular N—H⋯O hydrogen bond forms a six-membered ring and enforces an almost coplanar conformation for the acetamido group, the central benzene ring and the bridging carbonyl C—C(=O)—C group: the dihedral angles between the benzene ring and the acetamide and carbonyl C—C(=O)—C planes are 7.06 (11) and 7.17 (12)°, respectively. The dihedral angle between the two benzene rings is 67.43 (9)°. Because a strong hydrogen-bond donor is involved in the intra­molecular inter­action, the crystal packing is determined by weak C—H⋯O and C—H⋯Cl inter­actions.

## Related literature

The title compound is isostructural with 2-chloro­acetamido-5-chloro-2′-fluoro­benzophenone (Prasanna & Guru Row, 2000 ▶). For the isostructurality index, see: Kálmán *et al.* (1991 ▶); Kubicki & Szafrański (1998 ▶). For a related structure, see: Malathy Sony *et al.* (2005 ▶). For the biological activity of benzophenone derivatives, see: Evans *et al.* (1987 ▶). For a description of the Cambridge Structural Database, see: Allen (2002 ▶).
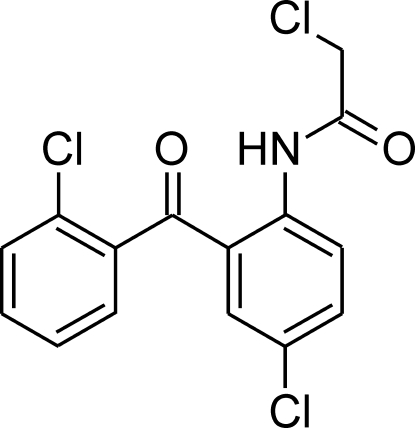


## Experimental

### 

#### Crystal data

C_15_H_10_Cl_3_NO_2_
*M* *_r_* = 342.59
Triclinic, 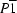

*a* = 7.5776 (9) Å
*b* = 10.1565 (10) Å
*c* = 10.7862 (12) Å
α = 70.069 (8)°
β = 77.604 (9)°
γ = 70.388 (8)°
*V* = 730.47 (14) Å^3^
*Z* = 2
Cu *K*α radiation
μ = 5.71 mm^−1^
*T* = 295 K
0.35 × 0.2 × 0.2 mm

#### Data collection

Oxford Diffraction SuperNova (single source at offset) Atlas diffractometer
Absorption correction: multi-scan (*CrysAlis PRO*; Oxford Diffraction, 2009 ▶) *T*_min_ = 0.117, *T*_max_ = 0.319
5245 measured reflections
2917 independent reflections
2610 reflections with *I* > 2σ(*I*)
*R*_int_ = 0.017

#### Refinement

*R*[*F* ^2^ > 2σ(*F* ^2^)] = 0.043
*wR*(*F* ^2^) = 0.113
*S* = 1.05
2917 reflections
230 parameters
All H-atom parameters refined
Δρ_max_ = 0.37 e Å^−3^
Δρ_min_ = −0.44 e Å^−3^

### 

Data collection: *CrysAlis PRO* (Oxford Diffraction, 2009 ▶); cell refinement: *CrysAlis PRO*; data reduction: *CrysAlis PRO*; program(s) used to solve structure: *SIR92* (Altomare *et al.*, 1993 ▶); program(s) used to refine structure: *SHELXL97* (Sheldrick, 2008 ▶); molecular graphics: *Stereochemical Workstation Operation Manual* (Siemens, 1989 ▶); software used to prepare material for publication: *SHELXL97*.

## Supplementary Material

Crystal structure: contains datablocks I, global. DOI: 10.1107/S1600536810003375/is2519sup1.cif

Structure factors: contains datablocks I. DOI: 10.1107/S1600536810003375/is2519Isup2.hkl

Additional supplementary materials:  crystallographic information; 3D view; checkCIF report

## Figures and Tables

**Table 1:** Hydrogen-bond geometry (Å, °)

*D*—H⋯*A*	*D*—H	H⋯*A*	*D*⋯*A*	*D*—H⋯*A*
N8—H8⋯O1	0.84 (3)	1.95 (3)	2.634 (2)	138 (3)
C6—H6⋯Cl14^i^	0.94 (3)	2.87 (3)	3.675 (2)	143 (2)
C11—H11*B*⋯O10^ii^	0.93 (3)	2.50 (3)	3.320 (3)	147 (2)
C18—H18⋯Cl14^iii^	0.95 (3)	2.84 (3)	3.745 (3)	161 (2)

Symmetry codes: (i) 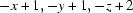; (ii) 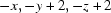; (iii) 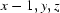.

**Fig. 1.**
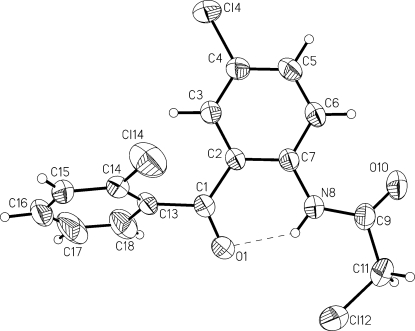
Anisotropic ellipsoid representation of the title compound together with atom labelling scheme. The ellipsoids are drawn at 50% probability level, hydrogen atoms are depicted as spheres with arbitrary radii. The intramolecular hydrogen bond is shown as a dashed line.

**Fig. 2.**
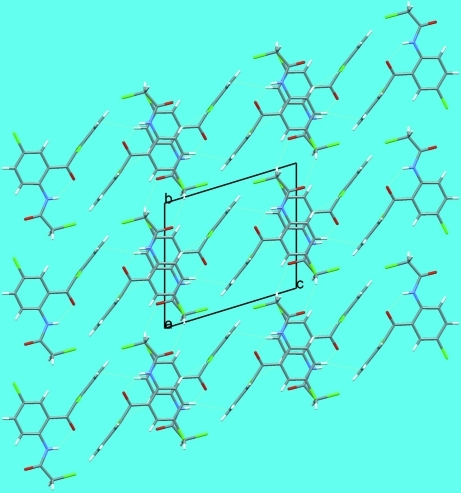
The crystal packing of the title compound, as seen along the a axis. Hydrogen bonds are shown as dashed lines.

## supplementary crystallographic information

### Comment

Benzophenone and related compounds have been reported to act as e.g., antiallergic, anti-inflammatory, antiasthmatic, antimalarial, anti-microbial and antianaphylactic agents (Evans *et al.*, 1987). Here we report the crystal structure of 2-chloroacetamido-5-chloro-2'-chlorobenzophenone (alternative name: 2-chloro-*N*-{4-chloro-2-[(2-chlorophenyl)carbonyl]phenyl}acetamide; 1, Scheme 1), which is an intermediate in the synthesis of certain anxiolytic, anticonvulsant and sedative drugs, and is also a starting material for the synthesis of diazepam and other benzodiazepines.

The structure of 1 is isostructural with the previously described 2-chloroacetamido-5-chloro-2'-fluorobenzophenone derivative (Prasanna & Guru Row, 2000). Both compounds crystallize in the triclinic P-1 space group, and the unit cell dimensions are similar. Also the positions of the atoms in the unit cell are similar, after applying the transformation (index 1 refers to Prasanna & Guru Row, 2 - to the present structure): x_2_=x_1_+0.5, y_2_=y_1_+0.5, z_2_=z_1_+1.5. The isostructurality index (Kálmán *et al.*, 1991), which describes the differences between the positions of the atoms in the unit cell has the value of 97.4% (for perfect isostructurality it should be 100%). Kubicki & Szafrański (1998) proposed the modification of this latter parameter which takes into account the point group symmetry and gives more absolute measure of the degree of isostructurality: it should be 1 for ideally isomorphous compounds and 0 for randomly distributed atoms. The value of this modified index is 0.94.

The conformation of 1 (Fig. 1) might be described by dihedral angles between four approximately planar fragments: acetamide [A, planar within 0.0065 (19) Å, with Cl2 atom significantly, by 0.110 (5)Å out of the plane], central phenyl [B, maximum deviation 0.0034 (14) Å], bridging carbonyl group C—C(=O)-C [C, max. deviation 0.015 (2) Å], and terminal phenyl [D, 0.0045 (19) Å]. The first three are close to coplanarity, the dihedral angles are A/B 7.06 (11)° and B/C 7.17 (12)°. Such a coplanar conformation of phenyl and carbonyl plane is quite uncommon for benzophenones, in a majority of the compounds found in the CSD (Allen, 2002) both phenyl rings are almost equally, and significantly, twisted with respect to the central plane. In the case of 1, as in some similar cases (for instance in the isostructural 2'-fluoro derivative but also in two crystal forms of 2-chloroacetamido-5-chlorobenzophenone (monoclinic: Prasanna & Guru Row, 2000, and triclinic: Malathy Sony *et al.*, 2005), the factor responsible for such a coplanar conformation is the intramolecular hydrogen bond N—H···O (cf. Table 1). This hydrogen bond closes the six-membered ring, planar within 0.072 (7) Å. The second phenyl ring, which has no factor that can stabilize coplanar conformation, is typically, by 62.14 (10)°, twisted with respect to the bridge. The Cl atom of chloroacetamide group is anti with respect to the oxygen atom [O10—C9—C11—Cl12 torsion angle of 176.4 (2)°] and syn with respect to the N atom [N8—C9—C11—Cl12 is -4.8 (3)°]

In the crystal structure there are some weak C—H···O and C—H···Cl interactions, which might be of some importance when the strong hydrogen bond donor is involved in intramolecular interaction (Fig. 2)

### Experimental

The title compound was obtained as a gift sample from R. L. FineChem, Bangalore, India. The compound was used without further purification. X-ray quality crystals were obtained by slow evaporation from ethyl acetate solution (m.p. 436-438 K).

### Refinement

Hydrogen atoms were found in the subsequent difference Fourier maps, and freely refined.

### Crystal data

**Table:**

C_15_H_10_Cl_3_NO_2_	*Z* = 2
*M**_r_* = 342.59	*F*(000) = 348
Triclinic, *P*1	*D*_x_ = 1.558 Mg m^−^^3^
Hall symbol: -P 1	Cu *K*α radiation, λ = 1.54178 Å
*a* = 7.5776 (9) Å	Cell parameters from 3832 reflections
*b* = 10.1565 (10) Å	θ = 4.4–75.1°
*c* = 10.7862 (12) Å	µ = 5.71 mm^−^^1^
α = 70.069 (8)°	*T* = 295 K
β = 77.604 (9)°	Prism, pink
γ = 70.388 (8)°	0.35 × 0.2 × 0.2 mm
*V* = 730.47 (14) Å^3^	

### Data collection

**Table:**

Oxford Diffraction SuperNova (single source at offset) Atlas diffractometer	2917 independent reflections
Radiation source: SuperNova (Cu) X-ray Source	2610 reflections with *I* > 2σ(*I*)
mirror	*R*_int_ = 0.017
Detector resolution: 10.5357 pixels mm^-1^	θ_max_ = 75.2°, θ_min_ = 4.4°
ω–scan	*h* = −9→9
Absorption correction: multi-scan (*CrysAlis PRO*; Oxford Diffraction, 2009)	*k* = −12→12
*T*_min_ = 0.117, *T*_max_ = 0.319	*l* = −13→13
5245 measured reflections	

### Refinement

**Table:**

Refinement on *F*^2^	Primary atom site location: structure-invariant direct methods
Least-squares matrix: full	Secondary atom site location: difference Fourier map
*R*[*F*^2^ > 2σ(*F*^2^)] = 0.043	Hydrogen site location: inferred from neighbouring sites
*wR*(*F*^2^) = 0.113	All H-atom parameters refined
*S* = 1.05	*w* = 1/[σ^2^(*F*_o_^2^) + (0.0551*P*)^2^ + 0.3949*P*] where *P* = (*F*_o_^2^ + 2*F*_c_^2^)/3
2917 reflections	(Δ/σ)_max_ = 0.001
230 parameters	Δρ_max_ = 0.37 e Å^−^^3^
0 restraints	Δρ_min_ = −0.44 e Å^−^^3^

### Special details

**Table:**

Geometry. All esds (except the esd in the dihedral angle between two l.s. planes) are estimated using the full covariance matrix. The cell esds are taken into account individually in the estimation of esds in distances, angles and torsion angles; correlations between esds in cell parameters are only used when they are defined by crystal symmetry. An approximate (isotropic) treatment of cell esds is used for estimating esds involving l.s. planes.
Refinement. Refinement of *F*^2^ against ALL reflections. The weighted *R*-factor wR and goodness of fit *S* are based on *F*^2^, conventional *R*-factors *R* are based on *F*, with *F* set to zero for negative *F*^2^. The threshold expression of *F*^2^ > 2σ(*F*^2^) is used only for calculating *R*-factors(gt) etc. and is not relevant to the choice of reflections for refinement. *R*-factors based on *F*^2^ are statistically about twice as large as those based on *F*, and *R*- factors based on ALL data will be even larger.

### Fractional atomic coordinates and isotropic or equivalent isotropic displacement parameters (Å^2^)

**Table:**

	*x*	*y*	*z*	*U*_iso_*/*U*_eq_	
C1	0.2330 (3)	0.4690 (2)	0.74281 (19)	0.0407 (4)	
O1	0.1250 (3)	0.58486 (19)	0.68930 (16)	0.0679 (5)	
C2	0.2780 (3)	0.4324 (2)	0.87982 (17)	0.0347 (4)	
C3	0.3816 (3)	0.2896 (2)	0.94118 (19)	0.0391 (4)	
H3	0.422 (3)	0.219 (2)	0.889 (2)	0.043 (6)*	
C4	0.4253 (3)	0.2515 (2)	1.06806 (19)	0.0419 (4)	
Cl4	0.55643 (9)	0.07373 (6)	1.14246 (6)	0.05951 (19)	
C5	0.3676 (3)	0.3534 (2)	1.1378 (2)	0.0482 (5)	
H5	0.393 (4)	0.325 (3)	1.228 (3)	0.074 (9)*	
C6	0.2645 (3)	0.4939 (2)	1.0804 (2)	0.0454 (5)	
H6	0.230 (4)	0.562 (3)	1.129 (3)	0.059 (7)*	
C7	0.2178 (3)	0.5364 (2)	0.95170 (18)	0.0358 (4)	
N8	0.1173 (3)	0.67993 (18)	0.88973 (17)	0.0415 (4)	
H8	0.094 (4)	0.696 (3)	0.812 (3)	0.067 (8)*	
C9	0.0393 (3)	0.7935 (2)	0.9426 (2)	0.0448 (5)	
O10	0.0375 (3)	0.78715 (18)	1.05679 (17)	0.0650 (5)	
C11	−0.0562 (4)	0.9388 (3)	0.8497 (3)	0.0545 (6)	
H11B	−0.006 (4)	1.010 (3)	0.851 (3)	0.066 (8)*	
H11A	−0.189 (5)	0.970 (4)	0.878 (3)	0.083 (10)*	
Cl12	−0.03818 (13)	0.94573 (7)	0.68136 (7)	0.0826 (3)	
C13	0.3127 (3)	0.3605 (2)	0.66429 (18)	0.0403 (4)	
C14	0.5017 (3)	0.3148 (2)	0.61956 (19)	0.0465 (5)	
Cl14	0.66554 (9)	0.37077 (10)	0.66357 (7)	0.0759 (2)	
C15	0.5645 (5)	0.2289 (3)	0.5339 (2)	0.0701 (9)	
H15	0.693 (6)	0.200 (4)	0.509 (4)	0.102 (12)*	
C16	0.4381 (7)	0.1877 (3)	0.4935 (3)	0.0855 (12)	
H16	0.478 (5)	0.129 (4)	0.433 (4)	0.109 (12)*	
C17	0.2507 (8)	0.2312 (4)	0.5360 (3)	0.0885 (12)	
H17	0.162 (6)	0.198 (4)	0.511 (4)	0.110 (13)*	
C18	0.1858 (5)	0.3185 (3)	0.6211 (3)	0.0646 (7)	
H18	0.054 (4)	0.345 (3)	0.647 (3)	0.064 (8)*	

### Atomic displacement parameters (Å^2^)

**Table:**

	*U*^11^	*U*^22^	*U*^33^	*U*^12^	*U*^13^	*U*^23^
C1	0.0457 (11)	0.0416 (10)	0.0345 (9)	−0.0059 (8)	−0.0076 (8)	−0.0154 (8)
O1	0.0874 (13)	0.0584 (10)	0.0450 (9)	0.0187 (9)	−0.0284 (8)	−0.0254 (8)
C2	0.0395 (9)	0.0372 (9)	0.0292 (8)	−0.0102 (8)	−0.0037 (7)	−0.0129 (7)
C3	0.0472 (11)	0.0362 (9)	0.0353 (9)	−0.0107 (8)	−0.0050 (8)	−0.0129 (8)
C4	0.0495 (11)	0.0382 (10)	0.0356 (9)	−0.0121 (8)	−0.0076 (8)	−0.0065 (8)
Cl4	0.0745 (4)	0.0419 (3)	0.0526 (3)	−0.0059 (3)	−0.0225 (3)	−0.0034 (2)
C5	0.0644 (14)	0.0501 (12)	0.0308 (9)	−0.0150 (10)	−0.0110 (9)	−0.0105 (9)
C6	0.0595 (13)	0.0464 (11)	0.0339 (10)	−0.0124 (9)	−0.0062 (9)	−0.0185 (9)
C7	0.0415 (10)	0.0362 (9)	0.0319 (9)	−0.0112 (8)	−0.0031 (7)	−0.0132 (7)
N8	0.0518 (10)	0.0364 (8)	0.0364 (9)	−0.0057 (7)	−0.0076 (7)	−0.0161 (7)
C9	0.0521 (12)	0.0401 (10)	0.0465 (11)	−0.0124 (9)	−0.0024 (9)	−0.0205 (9)
O10	0.0977 (14)	0.0495 (9)	0.0508 (9)	−0.0098 (9)	−0.0101 (9)	−0.0283 (8)
C11	0.0611 (15)	0.0390 (11)	0.0633 (14)	−0.0044 (10)	−0.0096 (12)	−0.0231 (10)
Cl12	0.1198 (6)	0.0491 (3)	0.0563 (4)	0.0106 (4)	−0.0247 (4)	−0.0128 (3)
C13	0.0556 (12)	0.0394 (10)	0.0296 (9)	−0.0123 (9)	−0.0095 (8)	−0.0129 (7)
C14	0.0579 (12)	0.0412 (10)	0.0317 (9)	0.0000 (9)	−0.0095 (9)	−0.0111 (8)
Cl14	0.0489 (3)	0.1181 (6)	0.0624 (4)	−0.0212 (4)	−0.0057 (3)	−0.0312 (4)
C15	0.101 (2)	0.0478 (13)	0.0343 (11)	0.0192 (14)	−0.0117 (13)	−0.0156 (10)
C16	0.172 (4)	0.0390 (12)	0.0384 (13)	−0.0081 (17)	−0.0247 (18)	−0.0161 (10)
C17	0.171 (4)	0.074 (2)	0.0541 (16)	−0.064 (2)	−0.033 (2)	−0.0190 (15)
C18	0.0825 (19)	0.0759 (17)	0.0535 (14)	−0.0381 (15)	−0.0126 (13)	−0.0231 (13)

### Geometric parameters (Å, °)

**Table:**

C1—O1	1.217 (3)	C9—O10	1.209 (3)
C1—C2	1.482 (2)	C9—C11	1.514 (3)
C1—C13	1.506 (3)	C11—Cl12	1.770 (3)
C2—C3	1.401 (3)	C11—H11B	0.93 (3)
C2—C7	1.417 (2)	C11—H11A	0.96 (3)
C3—C4	1.371 (3)	C13—C14	1.381 (3)
C3—H3	0.99 (2)	C13—C18	1.388 (3)
C4—C5	1.383 (3)	C14—C15	1.387 (3)
C4—Cl4	1.744 (2)	C14—Cl14	1.732 (2)
C5—C6	1.374 (3)	C15—C16	1.363 (5)
C5—H5	0.96 (3)	C15—H15	0.92 (4)
C6—C7	1.392 (3)	C16—C17	1.363 (6)
C6—H6	0.94 (3)	C16—H16	0.97 (4)
C7—N8	1.401 (3)	C17—C18	1.392 (4)
N8—C9	1.361 (3)	C17—H17	0.96 (4)
N8—H8	0.84 (3)	C18—H18	0.95 (3)
			
O1—C1—C2	122.49 (17)	N8—C9—C11	116.46 (19)
O1—C1—C13	116.25 (17)	C9—C11—Cl12	116.35 (15)
C2—C1—C13	121.21 (17)	C9—C11—H11B	109.2 (18)
C3—C2—C7	118.78 (17)	Cl12—C11—H11B	106.8 (18)
C3—C2—C1	118.91 (16)	C9—C11—H11A	111 (2)
C7—C2—C1	122.30 (17)	Cl12—C11—H11A	106 (2)
C4—C3—C2	120.57 (18)	H11B—C11—H11A	107 (3)
C4—C3—H3	122.2 (13)	C14—C13—C18	118.3 (2)
C2—C3—H3	117.3 (13)	C14—C13—C1	123.67 (19)
C3—C4—C5	120.60 (19)	C18—C13—C1	117.5 (2)
C3—C4—Cl4	120.01 (16)	C13—C14—C15	121.3 (3)
C5—C4—Cl4	119.38 (16)	C13—C14—Cl14	120.50 (15)
C6—C5—C4	120.02 (19)	C15—C14—Cl14	118.2 (2)
C6—C5—H5	119.4 (18)	C16—C15—C14	119.6 (3)
C4—C5—H5	120.5 (18)	C16—C15—H15	123 (2)
C5—C6—C7	120.93 (19)	C14—C15—H15	117 (2)
C5—C6—H6	118.3 (16)	C15—C16—C17	120.4 (2)
C7—C6—H6	120.8 (16)	C15—C16—H16	121 (2)
C6—C7—N8	121.99 (17)	C17—C16—H16	118 (2)
C6—C7—C2	119.09 (18)	C16—C17—C18	120.6 (3)
N8—C7—C2	118.90 (16)	C16—C17—H17	121 (2)
C9—N8—C7	128.23 (18)	C18—C17—H17	119 (3)
C9—N8—H8	116 (2)	C13—C18—C17	119.9 (3)
C7—N8—H8	116 (2)	C13—C18—H18	123.9 (17)
O10—C9—N8	125.6 (2)	C17—C18—H18	116.2 (17)
O10—C9—C11	117.95 (19)		
			
O1—C1—C2—C3	170.9 (2)	C7—N8—C9—O10	−3.3 (4)
C13—C1—C2—C3	−6.2 (3)	C7—N8—C9—C11	178.0 (2)
O1—C1—C2—C7	−8.3 (3)	O10—C9—C11—Cl12	176.4 (2)
C13—C1—C2—C7	174.60 (18)	N8—C9—C11—Cl12	−4.8 (3)
C7—C2—C3—C4	−0.6 (3)	O1—C1—C13—C14	114.9 (2)
C1—C2—C3—C4	−179.76 (19)	C2—C1—C13—C14	−67.8 (3)
C2—C3—C4—C5	0.1 (3)	O1—C1—C13—C18	−56.9 (3)
C2—C3—C4—Cl4	−179.37 (15)	C2—C1—C13—C18	120.4 (2)
C3—C4—C5—C6	0.4 (4)	C18—C13—C14—C15	−0.2 (3)
Cl4—C4—C5—C6	179.89 (18)	C1—C13—C14—C15	−171.9 (2)
C4—C5—C6—C7	−0.5 (4)	C18—C13—C14—Cl14	176.90 (18)
C5—C6—C7—N8	−178.2 (2)	C1—C13—C14—Cl14	5.2 (3)
C5—C6—C7—C2	0.1 (3)	C13—C14—C15—C16	−0.4 (3)
C3—C2—C7—C6	0.4 (3)	Cl14—C14—C15—C16	−177.56 (19)
C1—C2—C7—C6	179.63 (19)	C14—C15—C16—C17	0.5 (4)
C3—C2—C7—N8	178.80 (18)	C15—C16—C17—C18	0.1 (5)
C1—C2—C7—N8	−2.0 (3)	C14—C13—C18—C17	0.7 (4)
C6—C7—N8—C9	−5.3 (3)	C1—C13—C18—C17	172.9 (2)
C2—C7—N8—C9	176.4 (2)	C16—C17—C18—C13	−0.7 (4)

### Hydrogen-bond geometry (Å, °)

**Table:**

*D*—H···*A*	*D*—H	H···*A*	*D*···*A*	*D*—H···*A*
N8—H8···O1	0.84 (3)	1.95 (3)	2.634 (2)	138 (3)
C6—H6···Cl14^i^	0.94 (3)	2.87 (3)	3.675 (2)	143 (2)
C11—H11B···O10^ii^	0.93 (3)	2.50 (3)	3.320 (3)	147 (2)
C18—H18···Cl14^iii^	0.95 (3)	2.84 (3)	3.745 (3)	161 (2)

Symmetry codes: (i) −*x*+1, −*y*+1, −*z*+2; (ii) −*x*, −*y*+2, −*z*+2; (iii) *x*−1, *y*, *z*.
